# Current Sarcoidosis Models and the Importance of Focusing on the Granuloma

**DOI:** 10.3389/fimmu.2020.01719

**Published:** 2020-08-04

**Authors:** Landon W. Locke, Larry S. Schlesinger, Elliott D. Crouser

**Affiliations:** ^1^Department of Microbial Infection and Immunity, The Ohio State University Wexner Medical Center, Columbus, OH, United States; ^2^Host-Pathogens Interactions Program, Texas Biomedical Research Institute, San Antonio, TX, United States; ^3^Division of Pulmonary, Allergy, Critical Care, and Sleep Medicine, The Dorothy M. Davis Heart and Lung Research Institute, The Ohio State University Wexner Medical Center, Columbus, OH, United States

**Keywords:** sarcoidosis, granuloma, modeling, macrophages, lung

## Abstract

The inability to effectively model sarcoidosis in the laboratory or in animals continues to hinder the discovery and translation of new, targeted treatments. The granuloma is the signature pathological hallmark of sarcoidosis, yet there are significant knowledge gaps that exist with regard to how granulomas form. Significant progress toward improved therapeutic and prognostic strategies in sarcoidosis hinges on tractable experimental models that recapitulate the process of granuloma formation in sarcoidosis and allow for mechanistic insights into the molecular events involved. Through its inherent representation of the complex genetics underpinning immune cell dysregulation in sarcoidosis, a recently developed *in vitro* human granuloma model holds promise in providing detailed mechanistic insight into sarcoidosis–specific disease regulating pathways at play during early stages of granuloma formation. The purpose of this review is to critically evaluate current sarcoidosis models and assess their potential to progress the field toward the goal of improved therapies in this disease. We conclude with the potential integrated use of preclinical models to accelerate progress toward identifying and testing new drugs and drug combinations that can be rapidly brought to clinical trials.

## Introduction

Sarcoidosis is a complex immune disease with genetic susceptibility and environmental factors playing important roles. The disease occurs in about 15–40 per 100,000 people in the United States (1). In terms of both frequency and severity, the disease disproportionately affects women, people of African American and Northern European descent, and economically disadvantaged populations (1, 2). Severe clinical phenotypes are associated with progressive lung, heart, or brain damage and are debilitating and potentially fatal (2). The failure to effectively model sarcoidosis in the laboratory or in animals has been a roadblock to gaining crucial insights into the triggers and underlying cellular and molecular mechanisms driving the disease pathogenesis. This has manifested not only in terms of hindering the establishment of disease-specific biomarkers for diagnostic and prognostic purposes, but lack of mechanistic insight into disease regulating targets also impairs our capacity to treat this disease. In order to identify disease-specific treatments, the field needs a tractable model that recapitulates the essential elements of the disease pathogenesis. The granuloma is the signature pathological hallmark of sarcoidosis. Importantly, the architecture and immunology of granuloma formation differ significantly from one granulomatous disorder to the next. These differences, which are dictated by a highly complex and coordinated interplay of many diverse immune cell populations, are evident even during the early stages of granuloma formation, and they are further amplified as the structures evolve. However, a significant knowledge gap exists with regard to how granulomas form due to the fact that local factors, such as cytokines, chemokines, and direct cell-cell interactions among various immune cells, strongly influence the function of each immune cell during granuloma formation. *Significant progress toward improved therapeutic and prognostic strategies in sarcoidosis hinges on tractable experimental models that recapitulate the process of granuloma formation in sarcoidosis and allow for mechanistic insights into the molecular events involved*. The purpose of this review is to critically evaluate current sarcoidosis models and assess their potential to progress the field toward these goals. Selected sarcoidosis models described in the following sections have been summarized in Table 1.

**Table 1:** The strengths and limitations of current sarcoidosis research models.

**Research models of sarcoidosis granulomas**	**Strengths**	**Limitations**
Animal models	• Allow for therapeutic manipulation and preclinical testing of new therapeutics • Temporal information of disease progression • Genetically tractable	• Do not spontaneously form sarcoidosis (except for horses) • Granulomas are poorly reflective of human sarcoidosis • Not been well-validated against human diseased tissues
BAL cell-based models	• Cells interface with sarcoidosis-promoting environmental antigens • Cells are likely involved in the initial phases of the granulomatous response • Can establish disease biomarkers	• Cells are not engaged physically in granuloma formation • Continuous access to patients needed • Unstimulated immune cells do not recapitulate human sarcoidosis • Invasive to the patient
Unstimulated PBMC-based models	• Cells are directly involved in the formation of granulomas in the lungs • Can establish disease biomarkers	• Unstimulated immune cells do not recapitulate human sarcoidosis • Continuous access to patients needed
*In vitro* human granuloma model	• Accounts for the complex genetics dictating disease • Captures immune cell populations that engage in granuloma formation as well as cross-talk of these cells • Allows for pre-clinical testing of new therapies • Can accommodate the testing of potential disease-promoting pathogens and triggers • Can establish disease biomarkers	• Continuous access to patients needed • Limited ability to model fibrotic changes due to absence of tissue stromal elements • Lack of cell replenishment limits ability to track granulomas temporally (resolution vs. self-propagation)
Diseased tissues	• Gold standard for characterizing human sarcoidosis that is fully established in tissues	• Snapshots of established (i.e., late stage) disease • Not amenable to manipulation, limited insights into the early mechanisms of granuloma-genesis • Does not represent dynamic changes over time during evolution of sarcoidosis granulomas
Computational models	• Quickly and powerfully elucidate the dynamics and complex interplay of cells and mediators over time • Can be continuously improved as data becomes available • Can manipulate drug targets to model effects on granuloma formation and maintenance	• Needs companion cell-based models and human tissue validation to confirm model-derived hypothesis

## The Sarcoidosis Granuloma

Studies of diseased patient biopsy tissue are the foundation of sarcoidosis research. The original description of sarcoidosis in 1877 was based on the characteristic appearance of biopsied skin lesions and histological features that discriminated sarcoidosis from tuberculosis (TB) and other infectious granulomatous disorders (3). Despite the legitimacy of conducting research based on human sarcoidosis tissue samples, such research has yielded limited information regarding disease pathogenesis over the past 143 years. The information that can be obtained from sarcoidosis tissues is limited to a snapshot view of the disease in the form of established granulomas, a perspective that provides very limited insights into the critical and dynamic mechanisms underpinning the pathogenic formation of granulomas.

**Figure 1:**
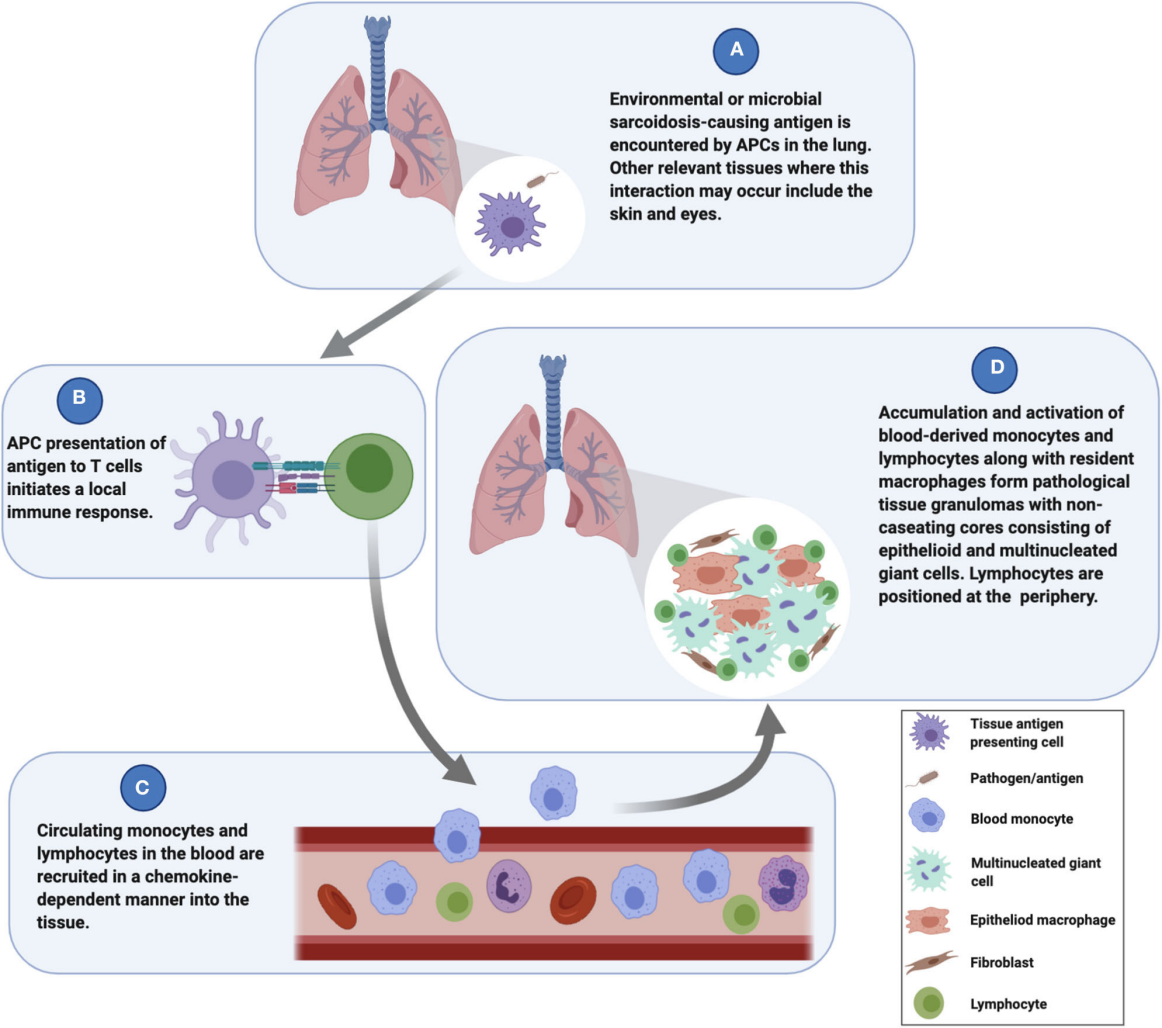
Mechanisms underlying the initiation and evolution of sarcoidosis granuloma formation.

Sarcoidosis granuloma formation is considered to be environmental antigen-mediated (4), and the lungs are the primary interface between environmental antigens and the host's immune surveillance system. This premise has led many researchers to investigate cells derived from bronchoalveolar lavage (BAL) fluid, particularly alveolar macrophages (AMs) and lymphocytes, to gain insight into sarcoidosis pathogenesis. Such studies indicated that sarcoidosis AMs exhibit enhanced antigen processing capacity and promote greater T cell activation (5), and as such, AMs likely play an important role during the initiation of the granulomatous response in the lungs. While it is likely that AMs interface with sarcoidosis-promoting environmental antigens and may be involved in the initial phases of the granulomatous response, they are not the primary source of macrophages involved in granuloma formation and are therefore insufficient for modeling their complexities. Cells in the peripheral blood, on the other hand, are directly involved in the formation of granulomas in the lungs (6) (Figure 1), and laboratory models based on peripheral blood mononuclear cells (PBMCs) have successfully replicated many of the histological and molecular features of human granulomatous diseases, including TB (7, 8), leprosy (9), and sarcoidosis (10). Along with tissue resident antigen-presenting cells (APCs) such as dendritic cells or AMs, blood monocytes act as sentinels for the detection of potential threats to the host and exit the vascular space to “patrol” within the interstitial space of the lungs (6). These monocytes will migrate back into the vascular space if no danger is sensed. When activated by a danger signal (e.g., bacterial antigens) (Figure 1A), the monocytes establish residency in the lung tissue (6, 11). The subsequent presentation of antigens by monocytes or tissue resident APCs to T cells (Figure 1B) leads to a localized immune response that drives the recruitment and activation of circulating monocytes and lymphocytes into the tissue (12, 13) (Figure 1C). The early granuloma begins to get assembled by these infiltrated cells along with tissue-resident cells to wall off the pathogen and protect the host (Figure 1D). Thus, PBMCs are essential for granuloma formation in the lungs and in other tissues as well.

## Animal Models of Sarcoidosis

Animal models are commonly relied upon to understand human disease mechanisms, and they offer potential strengths related to therapeutic manipulation and temporal information pertaining to disease progression. Currently there is no universally accepted animal model of human sarcoidosis, largely because animals [other than horses (14)] do not develop sarcoidosis and the link between human gene polymorphisms and disease prevalence has not been established to the degree that it can be recapitulated in the genetic manipulation of animals (e.g., mice) (15). While pulmonary granuloma formation can be achieved in rodents via pre-sensitization to putative antigens followed by pulmonary administration of the same antigens (i.e., a hypersensitivity response), injection and pulmonary embolization of pathogen-coated beads, or bronchial administration of foreign body particles, the granuloma cellular composition, morphology, local inflammatory milieu, and sustainability of these granulomas are poorly reflective of human sarcoidosis. For example, pulmonary granulomas that form in response to repeated exposures to the bacterium *Propionibacterium acnes* are consistent with hypersensitivity pneumonitis in that, unlike sarcoidosis granulomas, they are poorly formed, transient, and surrounded by prominent lymphocytic pneumonitis (16). Other models that instill carbon nanotubes into the airways of mice induce granulomas with features distinct from sarcoidosis, including foamy lipid-laden macrophages and a paucity of surrounding lymphocytes (17). The immunology of foreign body granulomas fundamentally differs from sarcoidosis because foreign bodies do not promenently feature adaptive immune responses (18, 19). Given the limited ability to recapitulate critical aspects of human sarcoidosis granulomas, animal models have not yielded major breakthroughs in the field of sarcoidosis, albeit with one exception.

A serendipitous link to human sarcoidosis granuloma formation was recently discovered based on a murine *Tsc2* knockout model (20). The *Tsc2* gene encodes for tuberous sclerosis protein-2 (TSC2), which normally represses the activity of mammalian target of rapamycin complex 1 (mTORc1) (21). The *Tsc2* knockout mice exhibit spontaneous systemic tissue granuloma formation with features resembling those of sarcoidosis patients with active disease progression, including epithelioid-like macrophages, M2 macrophage polarization, and mTORc1 pathway activation (20). However, genome wide association studies have not identified variations of the *Tsc2* gene as a risk factor for sarcoidosis, and the upstream regulators of mTORc1 in humans with sarcoidosis are unknown. Additionally, it is not known if abnormal regulation of mTORc1 alone is sufficient to cause disease in humans. In this regard, rapamycin, an mTORc1 inhibitor, is currently under investigation as a treatment for sarcoidosis and these studies may provide a more definitive answer to this question. It is reasonable to conclude based on available evidence that the murine *Tsc2* knockout model identifies an important immunological pathway that contributes to sarcoidosis disease progression. As such, the model could be leveraged for pre-clinical testing of new sarcoidosis therapeutics targeting mTORc1 regulation pathways.

A major barrier to developing a viable sarcoidosis model is the inability to identify a specific sarcoidosis disease-causing antigen. However, pulmonary sarcoidosis is clinically and histologically very similar to berylliosis, a human disease linked to genetic variability of T cell receptors for divalent beryllium molecules (22). Given the clinical similarities, it has been proposed that sarcoidosis and berylliosis may have similar mechanistic underpinnings (23, 24). Indeed, the berylliosis animal model produces multicellular aggregates of mononuclear cells in the lung consistent with human sarcoidosis (22). However, sarcoidosis patients do not exhibit abnormal T cell-mediated responses to beryllium (25). New data, discussed below, suggest that abnormal macrophage antigen presentation, as opposed to abnormal T cell-mediated antigen recognition, plays a primary role in sarcoidosis pathogenesis.

## Human Cell-Based Models of Sarcoidosis

Given that the polygenic nature of sarcoidosis renders it difficult to model using conventional murine knockout approaches, the field continues to rely on human subjects to conduct meaningful mechanistic research to understand sarcoidosis disease pathogenesis. There have been several studies that have examined single cell populations from BAL or blood of sarcoidosis patients, yielding new insights into the disease and potential disease trajectories. Drake et al. showed that CD4^+^ T cells derived from sarcoidosis BAL samples induced fibroblasts to produce increased amounts of collagen in a STAT3-IL17A dependent manner, thus clarifying signaling events upstream of fibrotic tissue changes (26). Given its ability to modify immune cell function, Yang et al. sought to identify DNA methylation changes in BAL cells from sarcoidosis patients ranging from remitting to severe phenotypes (27). While this study demonstrated an increased variability in DNA methylation in pooled sarcoidosis BAL cells, further insights into DNA methylation patterns associated with different sarcoidosis phenotypes are likely to emerge in the future from a larger patient sample size. In order to better clarify inciting antigens in sarcoidosis, Grunewald et al. used elegant molecular simulations based on CD4^+^ T cells derived from BAL to demonstrate a potential role for an autoantigen in triggering sarcoidosis (28). Other studies have similarly implicated an autoantigen in sarcoidosis. Chen et al. showed increased levels of serum amyloid A, an amyloid precursor protein, in biopsied sarcoidosis tissues, specifically showing it to be localized in macrophages and giant cells in granulomas (29). The authors postulated that this autoantigen might act to sustain granulomatous inflammation even if the inciting antigen was microbial in nature. In all, studies that have focused on single cell populations or diseased tissues have revealed factors that are likely important modifiers of disease but possess shortcomings with respect to determining the complex and multi-faceted mechanisms underlying pathogenic granuloma formation.

**Figure 2:**
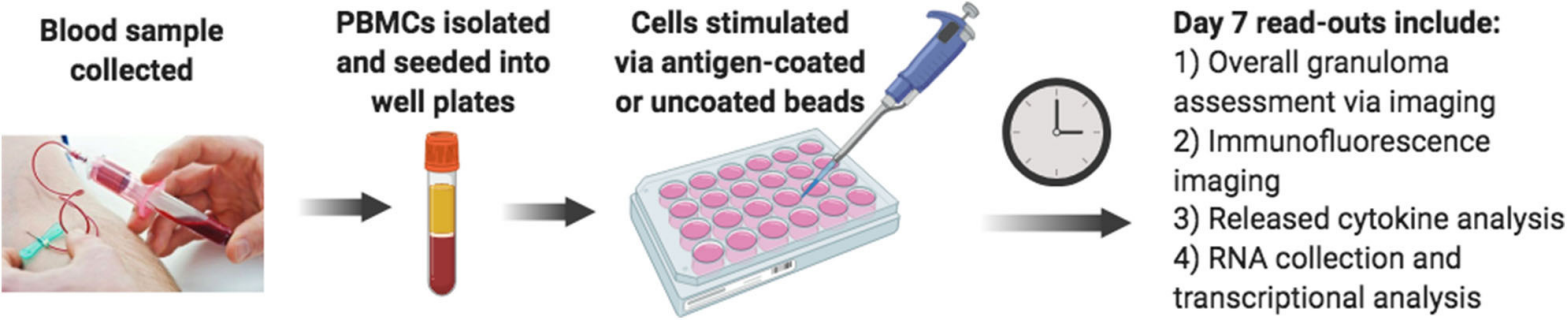
Schematic diagram of the *in vitro* human granuloma model.

Considering the limitations of current laboratory models, we sought to develop an improved lab model of human sarcoidosis, an *in vitro* human granuloma model (Figure 2), with the purpose of gaining mechanistic insights into the molecular events specifically involved in pathological granuloma formation. We posit that there are three key criteria of a viable model to recapitulate the process of granuloma formation in sarcoidosis. First, the model must account for the complex genetics and related unique immune features of sarcoidosis patients. Second, the model must allow for antigen-immune cell interplay, capturing the currently poorly defined immune cell populations that become dysregulated and engaged in granuloma formation. Third, the model must account for the immune cell microenvironment consisting of cytokines and chemokines that promote antigen processing, presentation, and immune cell phenotype changes that occur during the evolution of granulomas. The use of a stimulating antigen is crucial as there is a wealth of evidence supporting an evolution from active granulomatous infection phase to sterile sarcoidosis granulomas (30–32) often harboring non-viable microbial remnants of infectious organisms (33, 34). Recent *in vitro* granuloma models for TB have been developed that appear to exhibit many of these features (35).

**Figure 3:**
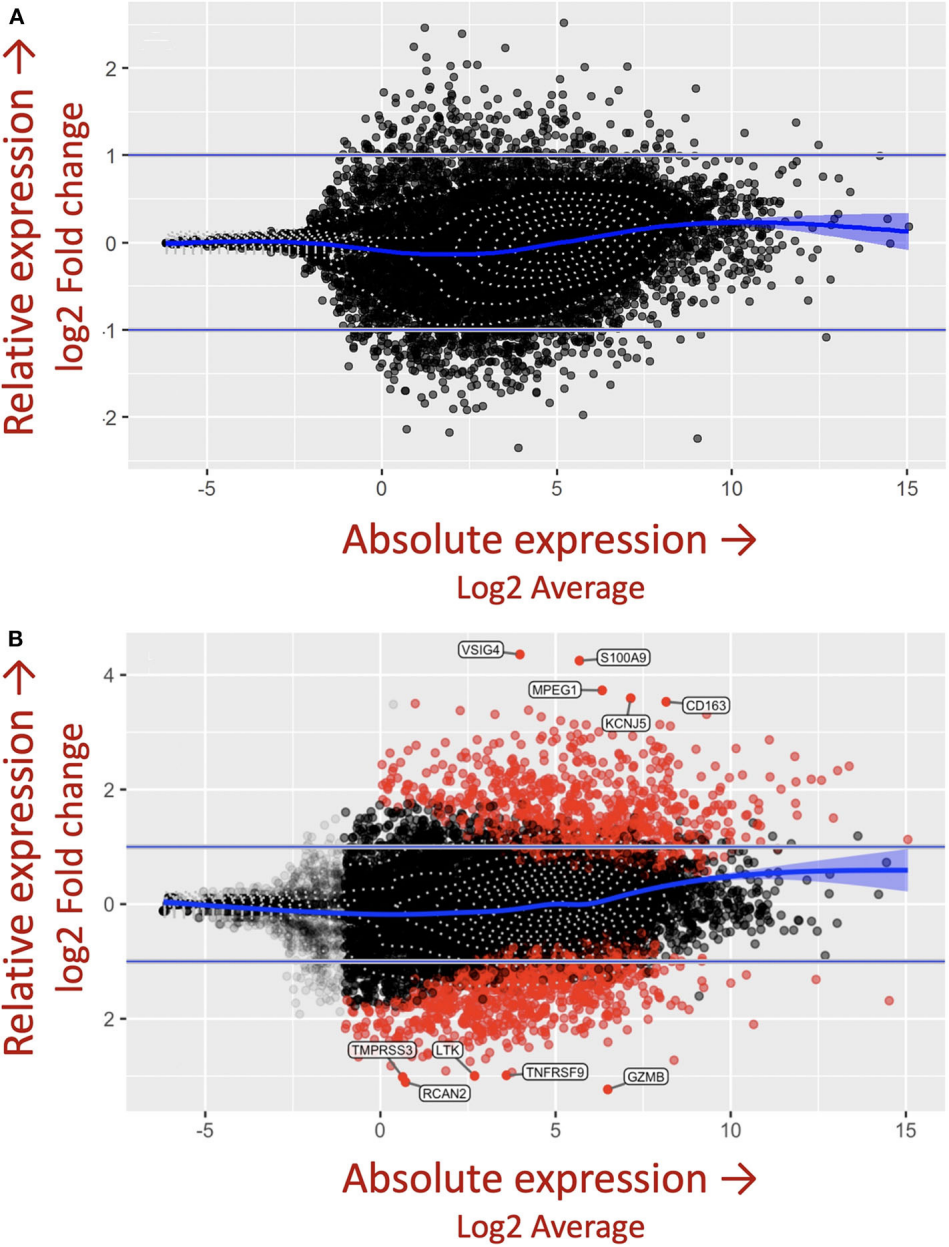
*M.tb* antigen stimulation induces a unique and divergent transcriptional response in sarcoidosis. Differential gene expression in PBMCs derived from patients with sarcoidosis and healthy control subjects after **(A)** uncoated and **(B)** *M.tb* antigen-coated bead stimulation shown as mva style plots. The x-axis is the log2 average of the gene expression level. All genes with an adjusted *P*-value of 0.05 and at least a log2-fold change in the magnitude of gene expression (indicated by the two horizontal blue lines) between *M.tb* antigen and uncoated beads are shaded red.

**Figure 4:**
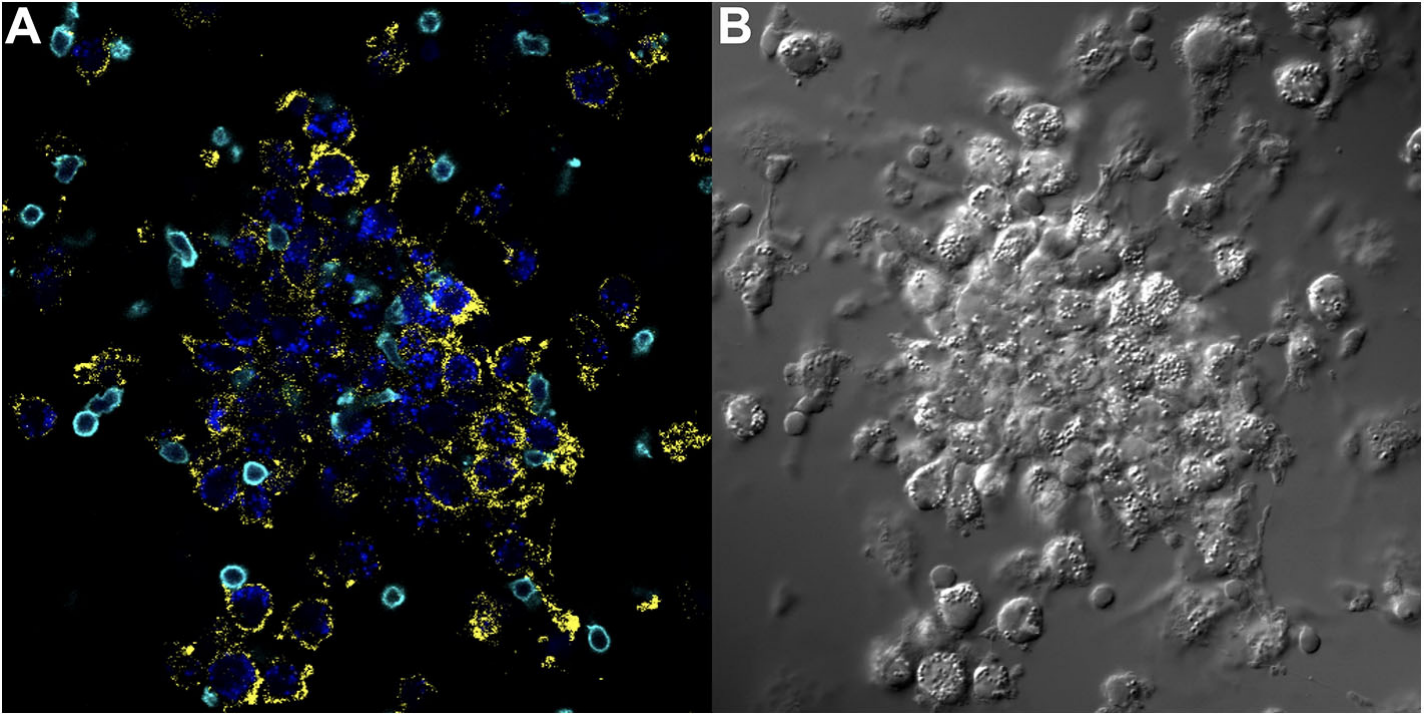
Key immune cell populations are represented in the *in vitro* human granuloma model. **(A)** Immunofluorescence microscopy image showing CD11b^+^ macrophages and CD3^+^ lymphocytes are present in a representative granuloma-like structure formed by *M.tb*. antigen-simulated PBMCs from a *M.tb*. naïve sarcoidosis patient. The image is a composite of 3 fluorescent channels: blue, yellow, and cyan channels represent the *M.tb*. antigen-coated beads, CD11b staining, and CD3 staining, respectively. **(B)** A differential interference contrast image of the same granuloma-like structure.

The importance of the criteria described above, including accounting for the complex genetics, immune cell cross-talk, and antigen-immune cell interplay dictating granuloma formation was demonstrated in the *in vitro* granuloma model by comparing PBMC responses between *Mycobacterium tuberculosis* (*M.tb*) naïve sarcoidosis patients and healthy controls. No difference in PBMC gene expression profiles was observed between *M.tb* naïve sarcoidosis patients and healthy controls at baseline (Figure 3A) (i.e., in the absence of antigen stimulation) in the model, but a vastly divergent response was noted in sarcoidosis PBMCs following challenge with immunogenic *M.tb* antigens (purified protein derivative, PPD) as reflected by >1,000 differentially expressed (DE) genes (Figure 3B). While the lack of DE gene expression observed in the absence of antigen likely speaks to variability of gene expression among humans, it provides confidence that the DE expression of >1,000 genes observed after antigen stimulation is not simply due to differences that were already apparent at baseline. Compared to healthy controls, *M.tb* antigen-stimulated sarcoidosis PBMCs also formed more robust granuloma-like aggregates composed of CD11b^+^ macrophages and CD3^+^ lymphocytes physically arranged in a manner typical of sarcoidosis granulomas (Figure 4) and produced a very different cytokine profile (10). These results challenge the relevance of studying immune cells in an unstimulated fashion and are consistent with the notion that sarcoidosis is a disease incited by exposure to infectious antigen (36).

## Modeling Divergent Mechanisms of Granuloma Formation Between Sarcoidosis and TB

Through its inherent representation of the complex genetics underpinning immune cell dysregulation, the *in vitro* granuloma model holds promise in providing detailed mechanistic insight into sarcoidosis–specific disease regulating pathways at play during early stages of granuloma formation. This was demonstrated through the common stimulation of PBMCs derived from *M.tb* naïve sarcoidosis patients and individuals with latent TB infection (LTBI) with *M.tb*-derived antigens. Both groups produced granuloma-like structures following stimulation, albeit the structures derived from LTBI individuals had a denser lymphocytic cuff. However, gene expression differences were vast between the groups revealing divergent mechanisms of granuloma formation that once again were dependent on antigen stimulation (37). Specifically, we found >5,000 unique DE genes in sarcoidosis compared to LTBI, with many of these DE genes being associated with macrophages (37). Ingenuity Pathway Analysis showed that one distinct pathway in sarcoidosis involves enhanced and prolonged antigen uptake, processing, and presentation (37). Featured in this pathway is the increased expression of vacuolar H^+^-ATPase (V-ATPase), a multisubunit proton pump that actively acidifies lysosomal compartments. In addition to this role, V-ATPase forms a signaling complex with mTORc1 that promotes its activation when phagolysosomes are acidified (38). This finding in the *in vitro* granuloma model links macrophage antimicrobial activities with abnormally sustained mTORc1 signaling shown to lead to unchecked granuloma formation in the *Tsc2* knockout mouse model and raises the speculation that evolution of sarcoidosis involves unchecked development of a granulomatous response as a result of enhanced microbial killing. The abnormal immune response in sarcoidosis featuring enhanced intracellular microbial killing via phagolysosomes is unlikely to be confined to *M.tb* and its antigens produced, as other intracellular pathogens, such as *Histoplasmosis capsulatum* and *P. acnes* have been incriminated in the pathogenesis of sarcoidosis (39).

## Modeling Macrophage Responses in Sarcoidosis

**Figure 5:**
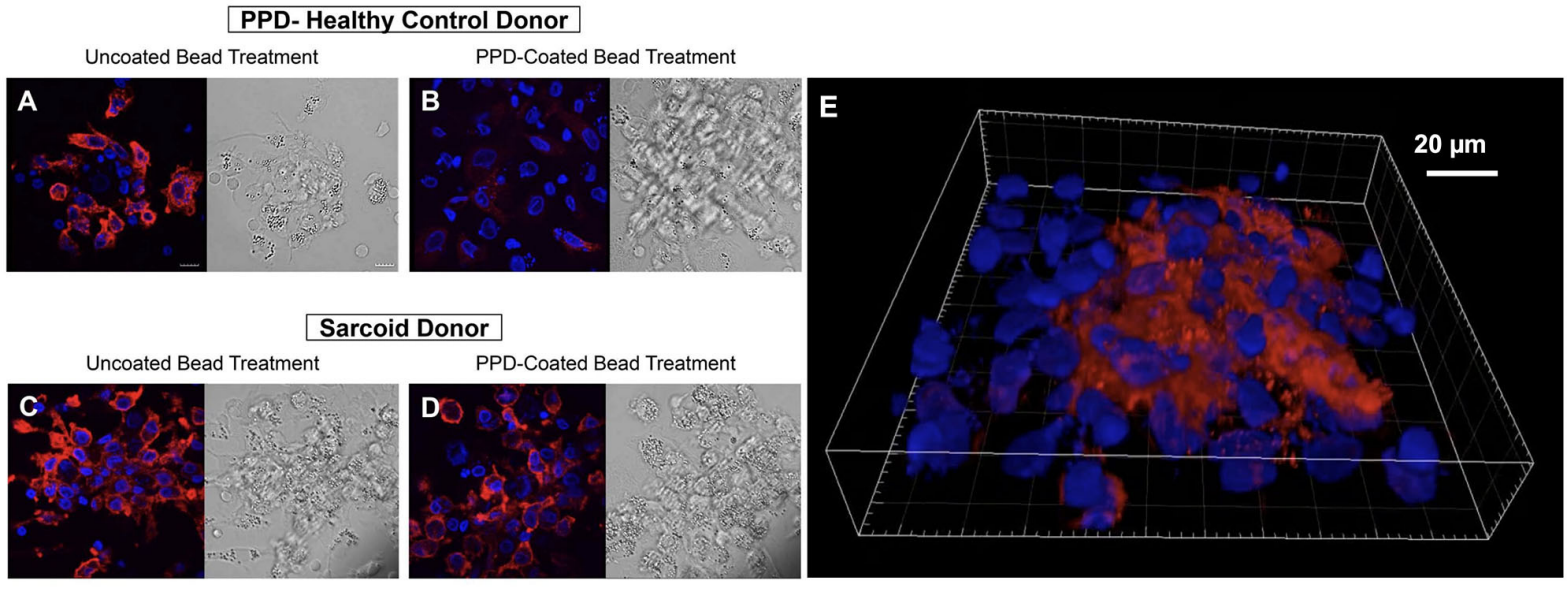
CD163 expression is increased in granuloma-like structures formed by antigen-stimulated sarcoidosis PBMCs in the *in vitro* human granuloma model. Immunofluorescence microscopy imaging demonstrating macrophage CD163 expression upon stimulating healthy control PBMCs with uncoated beads **(A)** with dramatic loss of expression following 7-days *M.tb* antigen-stimulation **(B)**. In contrast, abundant CD163 expression was observed on sarcoidosis macrophages after uncoated bead treatment **(C)** that persisted after *M.tb* antigen-stimulation **(D)**. A 3D rendered volume of a sarcoidosis granuloma-like structure 7 days after *M.tb* antigen-stimulation showing centrally clustered CD163-expressing macrophages **(E)**. DAPI and CD163 staining shown in blue and red, respectively.

**Figure 6:**
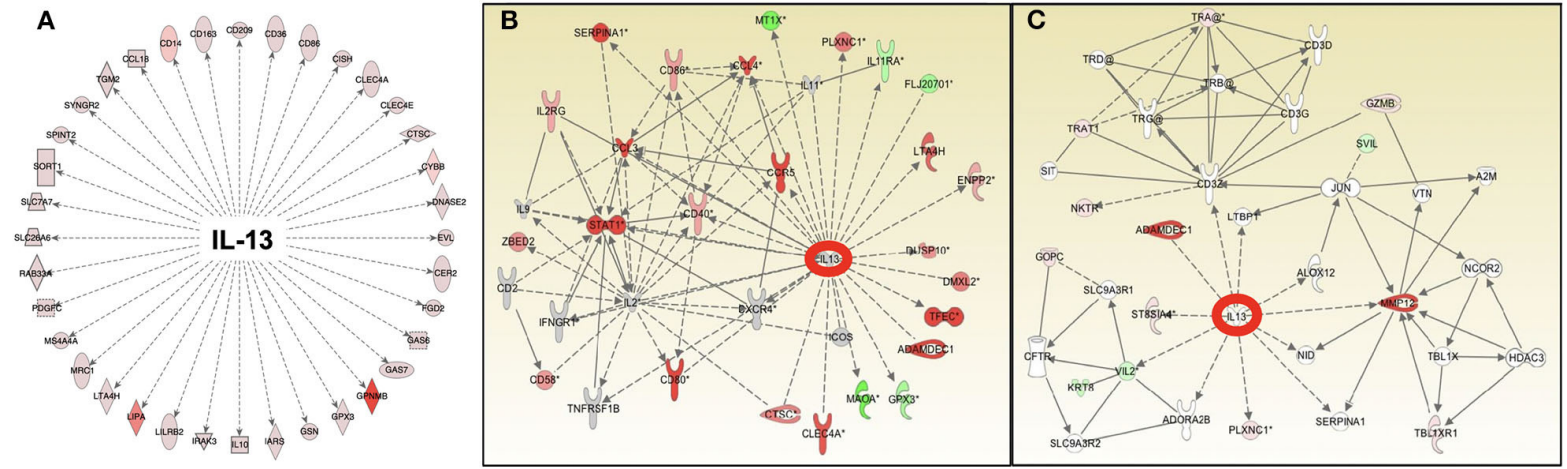
Pathway analysis of gene expression identifies IL-13 as an important and common upstream mediator of the antigen-dependent sarcoidosis granulomatous response in the *in vitro* granuloma model **(A)**, human sarcoidosis lymph node tissue **(B)**, and lung biopsies **(C)**.

Given that macrophages are the dominant cell type observed in sarcoidosis granulomas, there have been many different efforts aimed at modeling the macrophage response in sarcoidosis. Macrophage polarization is thought to play a major role in inflammatory diseases including diseases infectious in nature (7), and a growing body of evidence points to a functional imbalance in sarcoidosis in favor of a Th2-biased immune response. Macrophages display enormous plasticity in their phenotypes and appear along a continuum (40). One polar subset identified early on by defined agonists is the so-called M2-type macrophage that takes on immunoregulatory and tissue maintenance and reparative properties (41). Elevated expression of M2 macrophage-associated markers have been noted in diseased sarcoidosis tissues, including CD206 and CD163, and their expression correlated with disease severity (42–44). Macrophage features observed in our *in vitro* human granuloma model strongly align with this theory, including a significant increase in >50 different M2-like macrophage gene transcripts and elevated expression of CD163 in sarcoidosis granulomas at the protein level (Figure 5). Pathway analysis predicted the cytokine IL-13 as being an important upstream regulator of the observed gene expression changes in sarcoidosis compared to healthy controls after antigen stimulation, and we verified this gene network to be highly overrepresented in human sarcoidosis lung and lymph node tissues (45) (Figure 6). IL-13 signaling is associated with a Th2-biased immune response and is a known promoter of alternative or M2-like macrophage activation (46, 47). Furthermore, characteristic features of sarcoidosis such as multinucleated giant cell formation (48, 49) and fibrotic tissue changes (8, 39) associated with severe disease phenotypes are signature behaviors of M2-like macrophages.

Sarcoidosis and TB appear to have similar triggers, potentially occupying extremes of a common disease spectrum. Histologically, important differences between sarcoidosis and TB granulomas have been described with a subset of TB granulomas featuring lipid-containing, necrotic macrophages leading to caseation while sarcoidosis granulomas do not present with necrosis or caseation. CD163-expressing macrophages, which were recently shown to be highly abundant in sarcoidosis pulmonary granulomas but not in TB granulomas (43), do not accumulate lipids (50) and therefore their presence in sarcoidosis may explain why sarcoidosis granulomas are “non-caseating.” Examination of TB granulomas from infected macaques similarly revealed an absence of CD163 expression in caseating central cores of these structures containing lipid-filled macrophages (51). There is undoubtedly some overlap, however, in granuloma phenotypes between sarcoidosis and TB which is expected given the heterogeneity of these diseases. Ultimately, the connection between macrophage polarization, granuloma characteristics, and antimicrobial capacities including the restriction of *M.tb* growth remains incompletely understood, with the classic picture of M1 macrophages associated with improved protection likely an oversimplification.

Other data from the model that point to a functional imbalance in favor of Th2-immune signaling in sarcoidosis was the relatively blunted INF-γ production associated with sarcoidosis following *M.tb* antigen stimulation and the significant inhibition of granuloma formation in sarcoidosis through pharmacologic signal transducer and activator of transcription 6 (STAT6) inhibition (45). IL-13–mediated activation of STAT6 is a well-established pathway for M2-like macrophage polarization (52). It has also been shown that pharmacologic inhibition of STAT3 via upstream Janus kinase (JAK) inhibition leads to complete resolution of skin lesions in sarcoidosis patients (53). Activation of STAT3 is associated with M2-like macrophage polarization (54). In the *in vitro* human granuloma model we also observed a subset of STAT1-regulated genes that were DE between sarcoidosis and LTBI following *M.tb* antigen stimulation, suggesting a more complex signaling landscape. Indeed, the observed co-existence of both non-CD163-expressing macrophages and CD163-expressing macrophages in the granuloma-like structures (Figure 5) supports heterogeneous signaling at the level of the granuloma. Collectively, data from the model with respect to transcriptome-based network characteristics, immune signaling dysfunction, and macrophage immunohistological protein expression patterns reflective of diseased tissues indicate that antigen-stimulated PBMCs derived from patients with sarcoidosis has the ability to manifest the complex and, as yet, undefined genetic features that predispose to sarcoidosis.

## Mathematical Modeling of Sarcoidosis

**Figure 7:**
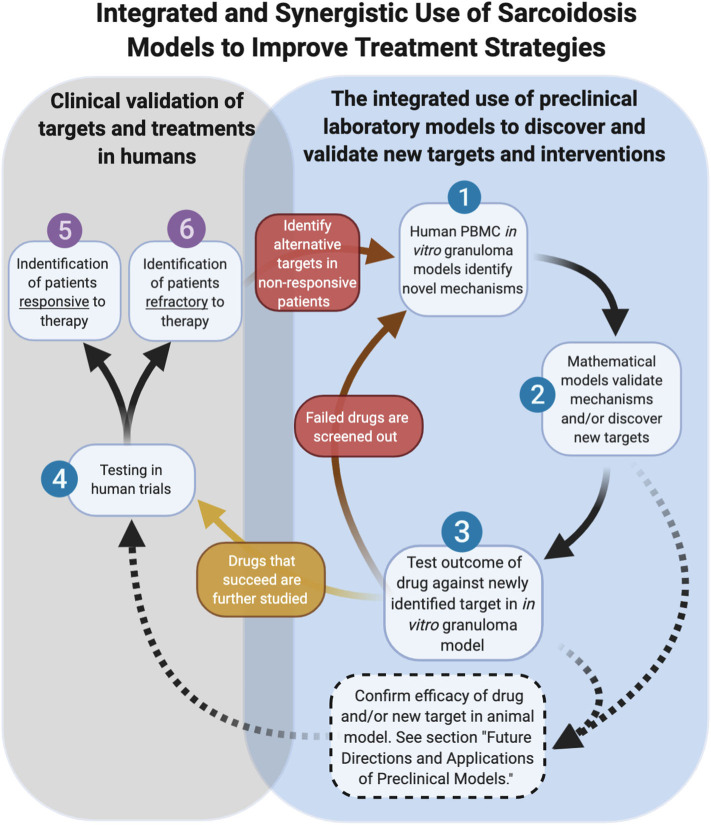
Integrated and synergistic use of sarcoidosis models. Dashed arrows represent future pathways dependent on animal model advancements.

As cell-based laboratory models continue to advance, the use of mathematical models will act in concert with them to accelerate the pace of discovery (55, 56). A computational model of sarcoidosis has been established and is capable of modeling physical measurements and parameters based on input from companion *in vitro* models and human studies to model the interconnectedness of the cytokine, chemokine, and growth factor proteins contributing to granuloma formation over time (57). We envision that the mathematical model can be used to rapidly interrogate the complex interplay of cells and inflammatory mediators on the evolution of granuloma formation over time through the manipulation of a variable (cytokine for example) or combination of variables, thus informing on specific druggable targets in sarcoidosis. Targets identified in this manner can be rapidly tested in the *in vitro* model for efficacy and thus be combined in a synergistic fashion to bring new therapies (mono or in combo with another) into human trials to effectively inhibit the cycle of pathological granuloma formation (Figure 7).

## Future Directions and Applications of Preclinical Models

As we look toward the future of therapeutic development in sarcoidosis, the role of animal models is currently unclear. Undoubtedly animal models have proven valuable for defining new aspects of disease pathogenesis and pre-clinical testing of new therapeutics. Relevant to sarcoidosis, novel imaging techniques can be performed in mice to enable powerful visual insights into the interplay of immune cells and tissue stroma (58). The future development of new transgenic animal models of sarcoidosis, however, will be contingent upon identifying disease-causing genetic factors in humans. An alternative approach would be to “humanize” animals, a process involving the replacement of the native bone marrow with stem cells from human sarcoidosis donors. There are inherent limitations of this approach, including impaired adaptive immunity in the humanized mice (59), which likely hinder these models from accurately modeling the full cellular and molecular determinants of granuloma formation in sarcoidosis. A humanized model of TB demonstrated pathology that closely modeled human disease, lending promise to this approach in the future (60). We envision a future role of humanized animal models of sarcoidosis that are explored in parallel with *in vitro* human granuloma models as complementary platforms for testing new therapies and evaluating pharmacokinetic and toxicity aspects ahead of human trials (Figure 7).

**Figure 8:**
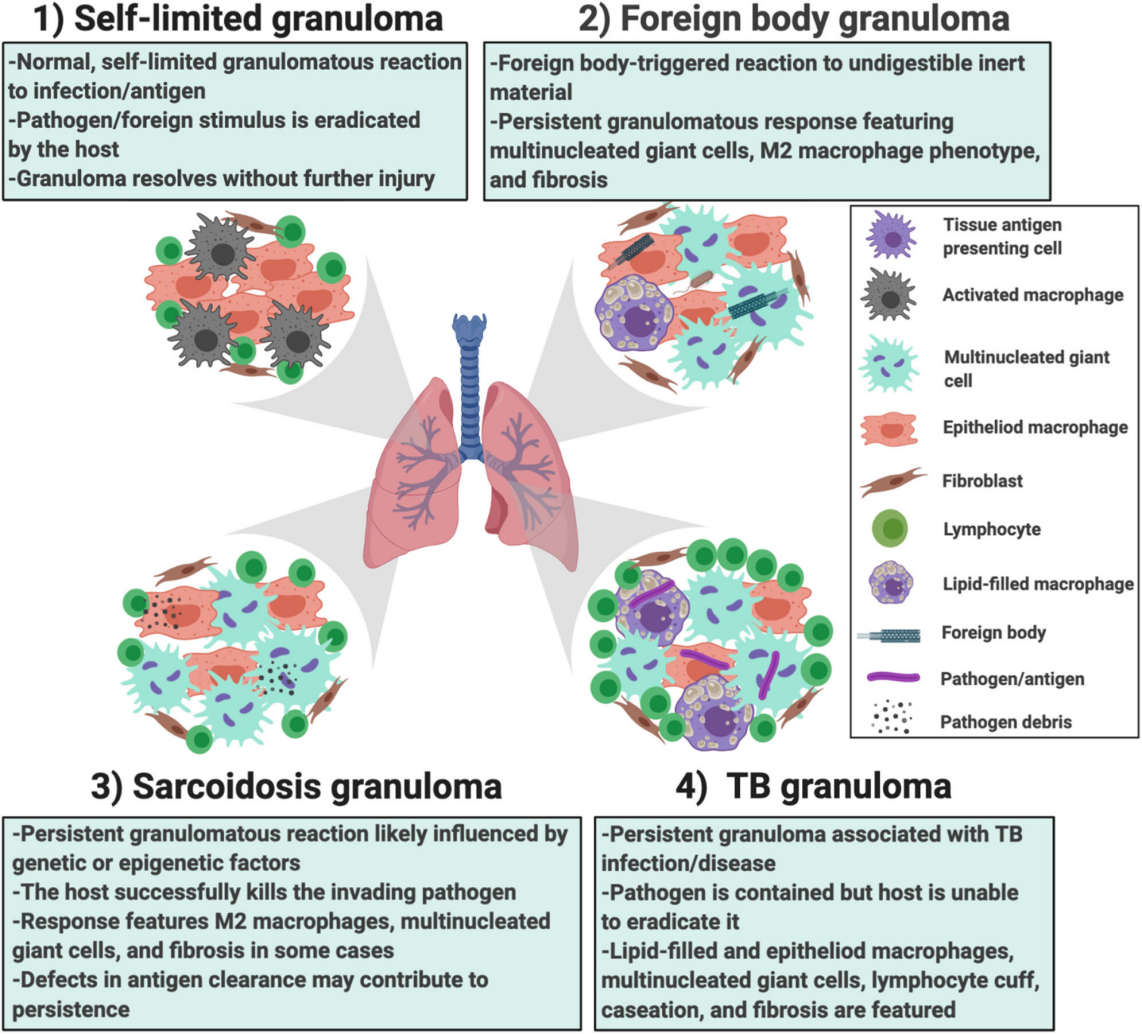
Different scenarios in which the *in vitro* human granuloma model can be used to better understand the dynamic, multicellular mechanisms underpinning distinct granulomatous immune responses that occur in humans ranging from (1) self-limited immune reactions; (2) foreign body immune reactions; (3) sarcoidosis granulomas; and (4) TB granulomas. The granuloma model can be used to test mechanistic hypotheses as well as further our understanding of the importance of environmental (e.g., the type of antigen used to promote granulomas) and host factors (e.g., genetics/race, epigenetic factors/sex, etc.) in these reactions. They can also be used for throughput testing of new potential therapies.

In addition to the providing greater insights into granuloma formation in sarcoidosis, the *in vitro* human granuloma model could be leveraged to screen novel therapies and determine the contribution of specific genes to granuloma formation using siRNA or CRISPR technology (Figure 7). Because the model accounts for the inherent genetic variables that predispose to disease, it could be used to gain insights into the ethnic/racial and gender disparities characteristic of this disease. Given that approximately one-third of patients have a progressive form of the disease (61), the model may be able to clarify the unique mechanisms driving granuloma formation in this severe phenotype. An additional strength of the *in vitro* human granuloma model is that it can be used to better understand the dynamic, multicellular mechanisms underpinning distinct granulomatous immune responses that occur in humans and is not restricted to sarcoidosis (Figure 8). For example, the model can be used to explore mechanisms driving early granulomatous responses to etiologic factors other than TB antigens, including exposures to foreign bodies.

There are some caveats of current *in vitro* cell culture models to address. For example, the *in vitro* human granuloma model is likely not capturing the entire spectrum of disease due to the absence of a tissue-mimicking environment. Recent advances in organ-on-a-chip technology are beginning to provide new opportunities in *in vitro* cell culture models such as the modeling of more complex cell interactions involving extracellular matrix components and stromal cells, although to date most of these models lack immune cells (62). Enabling the cross-talk between immune cells and matrix cells such as fibroblasts may allow for mechanisms driving fibrotic changes to be modeled in sarcoidosis, a signature feature of severe disease phenotypes (63). Organ-on-a-chip technology also allows for the continuous perfusion of cells, such as PBMCs, through a vascular-like system, thus potentially modeling cellular recruitment processes and enabling a more prolonged study of the eventual fate of granulomas (i.e., self-perpetuating vs. disaggregating). As with any modeling endeavor, it is challenging to know when a model is “good enough.” In the case of sarcoidosis, any modeling advancement must be weighed against the added cost, throughput potential, and the burden of validation.

## Conclusion

The failure to effectively model sarcoidosis in the laboratory or in animals continues to hinder the discovery and translation of new, targeted treatments. Preclinical models that are capable of clarifying the significant knowledge gap surrounding the triggers and mechanisms driving granuloma formation and self-perpetuation in sarcoidosis will be key to this cause. Compared to modeling approaches that study only a single cell type, the patient-derived PBMC-based *in vitro* granuloma model allows for the interrogation of antigen-immune cell interplay and related cell-cell interactions driving the various stages of granuloma formation in sarcoidosis and underpinned by the complex and, as yet, undefined genetic features of this disease. The *in vitro* human granuloma model provides a powerful approach to addressing critical knowledge gaps in sarcoidosis, and we envision its complementary use with other pre-clinical models will likely accelerate progress toward identifying and testing new drugs and drug combinations that can be rapidly brought to clinical trials.

## Author Contributions

EC, LS, and LL wrote the article. EC and LL contributed to designing the figures and table for the article. All authors contributed to the article and approved the submitted version.

## Conflict of Interest

The authors declare that the research was conducted in the absence of any commercial or financial relationships that could be construed as a potential conflict of interest.

## Abbreviations

AM: alveolar macrophage
BAL: bronchoalveolar lavage
PBMC: peripheral blood mononuclear cells
JAK: Janus kinase
STAT6: signal transducer and activator of transcription 6
V-ATPase: vacuolar H+-ATPase
*M.tb*: *Mycobacterium tuberculosis*
mTORc1: mammalian target of rapamycin complex 1
Tsc2: tuberous sclerosis-2 gene
DE: differentially expressed
TB: tuberculosis
LTBI: individuals with latent TB infection
PPD: purified protein derivative.
